# Named entity recognition of Chinese electronic medical records based on a hybrid neural network and medical MC-BERT

**DOI:** 10.1186/s12911-022-02059-2

**Published:** 2022-12-01

**Authors:** Peng Chen, Meng Zhang, Xiaosheng Yu, Songpu Li

**Affiliations:** 1 College of Computer and Information Technology, China Three Gorges University, Yichang, 443002 China; 2 Hubei Province Engineering Technology Research Center for Construction Quality Testing Equipments, China Three Gorges University, Yichang, 443002 China; 3 College of Economics and Management, China Three Gorges University, Yichang, 443002 China

**Keywords:** Named entity recognition, BERT model, Chinese electronic medical record, Hybrid neural network

## Abstract

**Background:**

Named entity recognition (NER) of electronic medical records is an important task in clinical medical research. Although deep learning combined with pretraining models performs well in recognizing entities in clinical texts, because Chinese electronic medical records have a special text structure and vocabulary distribution, general pretraining models cannot effectively incorporate entities and medical domain knowledge into representation learning; separate deep network models lack the ability to fully extract rich features in complex texts, which negatively affects the named entity recognition of electronic medical records.

**Methods:**

To better represent electronic medical record text, we extract the text’s local features and multilevel sequence interaction information to improve the effectiveness of electronic medical record named entity recognition. This paper proposes a hybrid neural network model based on medical MC-BERT, namely, the MC-BERT + BiLSTM + CNN + MHA + CRF model. First, MC-BERT is used as the word embedding model of the text to obtain the word vector, and then BiLSTM and CNN obtain the feature information of the forward and backward directions of the word vector and the local context to obtain the corresponding feature vector. After merging the two feature vectors, they are sent to multihead self-attention (MHA) to obtain multilevel semantic features, and finally, CRF is used to decode the features and predict the label sequence.

**Results:**

The experiments show that the F1 values of our proposed hybrid neural network model based on MC-BERT reach 94.22%, 86.47%, and 92.28% on the CCKS-2017, CCKS-2019 and cEHRNER datasets, respectively. Compared with the general-domain BERT-based BiLSTM + CRF, our F1 values increased by 0.89%, 1.65% and 2.63%. Finally, we analyzed the effect of an unbalanced number of entities in the electronic medical records on the results of the NER experiment.

## Introduction

An electronic medical record (EMR) is a text-based record of a patient's diagnosis and treatment process in hospitals and other medical institutions. It is typically stored in an unstructured format and includes information such as the patient's health status and symptoms, medications, diseases, and various test indicators. For example, in “患者缘于 20分钟前骑自行车被他人开车碰倒,伤及右膝关节、且右腕疼痛,局部肿胀且活动受限,无头痛头晕,无恶心呕吐,无意识障碍。急来我院门诊检查CT,右膝正侧位、右腕关节正侧位回报:未见异常。 [The patient was injured 20 min ago when her bicycle was knocked down by another person's car, which caused pain in the right knee and right wrist, localized swelling and limited movement, no headache or dizziness, no nausea or vomiting, and no impairment of consciousness. Urgently came to our outpatient clinic for CT examination, right knee ortholateral and right wrist ortholateral returns; no abnormality seen.],” “右膝关节 [right knee]” is a body part, “局部肿胀且活动受限 [localized swelling and limited movement]” is a disease symptom, and “检查CT [CT examination]” is a test. Such EMRs make it easier for medical institutions and specialists to analyze information about patients' conditions and provide treatment recommendations [1].

Named entity recognition (NER) is a critical task for extracting significant entities from text data [2]. Early NER was primarily performed through feature selection and model improvement methods and achieved some satisfactory results. Deep learning-based methods can automatically discover hidden features in text and obtain better results in NER tasks than feature-based methods. Therefore, many studies in recent years have concentrated on identifying named entities in EMR text using deep neural network-based methods. Since a single deep neural network model frequently fails to extract feature information from text well, it does not perform well in some data samples. To obtain a better text representation, researchers have proposed another series of word vector representation models, i.e., pretrained models. Deep learning methods for NER that are currently popular are typically pretrained models based on word embedding. One of the more practical pretraining models is the BERT model, which is based on the Transformer bidirectional encoder [3, 4]. It can pretrain corpora in various fields and obtain better word vector representations from the word and sentence context levels.

Furthermore, most downstream NLP tasks based on the BERT model can achieve good results [5]. BERT is also often applied as a pretraining model for information processing in Chinese medical texts [6]. However, due to the complexity of the text structure of medical EMRs, generic-domain pretraining models (such as BERT) cannot represent medical texts well, and medical domain-related pretraining models are required for word embedding representation of Chinese EMR texts. Similarly, because of the complexity of the text structure of medical EMRs, general pretrained BERTs do not represent medical text well, and a single downstream deep network model does not extract word vector features well. Therefore, in response to recent research, we make the following main contributions in this work:

1. We use a new pretraining MC-BERT model based on a Chinese clinical corpus, which enables us to express the word information related to the medical field. Our experimental results show that the model has high accuracy in NER tasks.
2. We introduce a new hybrid neural network model (BiLSTM + CNN + CRF) in the downstream model of MC-BERT for better extraction of vector features and decoding to obtain entity labels. The experimental results show that the hybrid model proposed in this paper improves in all evaluation metrics compared to the baseline model.
3. Finally, we analyze the effect of an unbalanced ratio of entities on NER in the experimental results of the hybrid model.

## Related work

There are primarily four methods for named entity recognition research: (1) rule-based methods, (2) statistics-based machine learning methods, (3) deep learning-based methods [7], and (4) named entity recognition using pretrained models.

Human-made rules are used in the rule-based NER approach. Rules can be created based on specific domains or grammar and word patterns. However, due to the specificity of different fields and the dictionary's incompleteness, such methods have high precision but low recall. Statistical machine learning methods can convert NER to classification or sequence labeling tasks using supervised learning, and this approach relies on the construction of features, such as in hidden Markov models (HMMs) and conditional random field models (CRFs) in probabilistic graphs [8, 9]. The designed features are then trained on annotated corpora to identify similar entities in unknown text. Although this method is significantly better than the rule-based method, it also necessitates many annotations by experts with professional domain knowledge, and the labor and time costs are high.

In recent years, deep learning methods have become mainstream in NER. The key advantage of deep learning is the capability of representation learning and the semantic composition empowered by both vector representation and neural processing [10]. Deep neural networks based on CNN, LSTM, or BiLSTM, combined with machine learning models such as CRF, are typical deep learning models for NER that can learn similar representations of semantically or functionally similar words and can effectively extract features from text data [11–13]. Zhang et al. proposed a grid LSTM model for NER on Chinese text. This model can add the meaning of the word itself to the word vector-based model, reducing the impact of Chinese word segmentation errors [14]. Tang et al. proposed an attention-based CNN + LSTM + CRF model. This model was used to identify entities in Chinese clinical texts and produced excellent NER results [15]. Deep learning-based methods can extract text features automatically using neural networks, discover hidden features, and update network model parameters end-to-end using gradient descent to optimize the model. However, the network model may overfit when the deep neural network is presented with training samples from a small corpus.

Researchers have investigated a number of word embedding pretraining models to improve the accuracy of deep learning named entity recognition. For example, the earliest word2vec and ELMo models were based on the LSTM structure [16, 17], and now pretraining models based on the Transformer structure have been proposed, such as GPT-3, ERNIE, and FLAT [18–20]. The emergence of training models is exceptionally beneficial for a variety of downstream NLP tasks. Pretraining models avoid training the model from scratch, significantly reducing the training time and preventing the deep neural network model from overfitting after training on a small sample dataset. Since Google proposed the BERT pretraining model in 2018, it has achieved good results in the representation of word vectors and has gradually gained popularity. Researchers have presented various domain-specific BERT models based on the BERT pretraining model. In the medical field, for example, Lee et al. introduced the first BERT pretraining model based on English medical text data, BioBERT, which was the first domain-specific language representation model pretrained on a large-scale biomedical corpus [21], but was not intended for Chinese medical text. Zhang et al. used the downstream model of BiLSTM + CRF to input features and pretrained a BERT model on a Chinese clinical text corpus to solve the problem of breast cancer entity recognition, but the authors did not publish their pretrained model [22]. Li et al. trained a BERT model by crawling a large number of Chinese medical-related web texts and released the PyTorch version of the model [23]. In recent research, Ali's team proposed a novel conceptualized representation learning method for adapting pretrained language models to the Chinese biomedical corpus, and this method pretrains MC-BERT models based on the Chinese biomedical domain [24]. This research presented the first pretrained BERT model using a large-scale Chinese biomedical domain corpus injected into representation-based learning. The team tested the effectiveness of the MC-BERT Chinese pretraining model on its newly published Chinese Biomedical Language Understanding Evaluation Benchmark (ChineseBLUE) dataset.

## Hybrid neural network model based on medical MC-BERT

Named entity recognition in Chinese EMRs is a sequence labeling task in natural language processing. The deep learning-based method effectively extracts text feature information and solves the problem of named entity recognition in EMRs. Some researchers are currently using the BERT pretraining model for named entity recognition research, such as the CNN model combined with BERT [25]. The pretrained model can more accurately represent the text's word embedding, resulting in a better-named entity recognition effect.

These methods have solved the named entity recognition problem of complex EMR texts in the medical field to some extent, but in practice, there are still pretrained models in the general field that cannot sufficiently represent Chinese EMR texts, and a single deep neural network cannot fully extract the feature information of the word vector in the text.

**Fig. 1:**
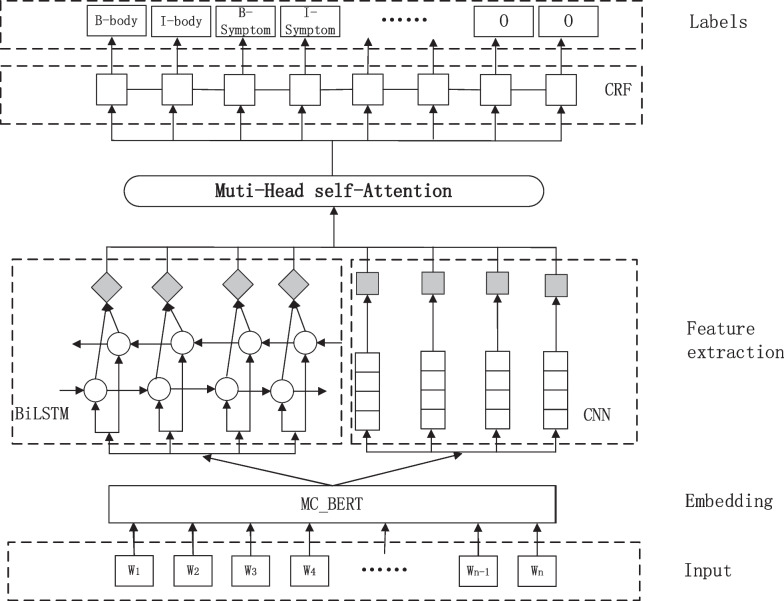
Architecture diagram of the hybrid neural network model based on MC-BERT

This paper constructs a hybrid neural network model based on medical MC-BERT to address these issues. The model includes an MC-BERT layer for word embedding, a BiLSTM layer, a CNN layer, a multihead self-attention (MHA) mechanism, and a CRF layer in the downstream model. Among these, MC-BERT is used for medical text word embedding, and Chinese characters are converted into word vectors with text information using MC-BERT to achieve a better embedding. The obtained embedded word vectors are then simultaneously fed into BiLSTM and multilayer CNN models, and feature extraction is performed on the word vectors. The output results of these two parts are fused, and the multihead self-attention mechanism is combined to extract global feature correlation information from multiple angles and levels. Finally, the CRF layer can fully consider the intercharacter tag dependencies and constraints and decode them using CRF to ensure the reasonableness of the final predicted tags. The architecture of the hybrid neural network model based on MC-BERT is shown in Fig. 1.

### BERT models

**Fig. 2:**
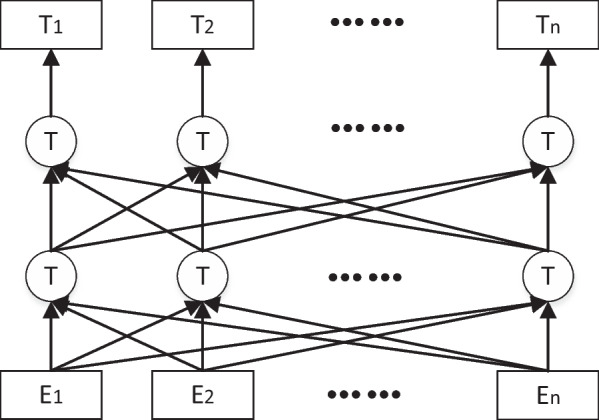
BERT model structure diagram (from Ref. [4])

BERT is an excellent pretraining model for text word vector representation. It is made up of a multilayer bidirectional Transformer encoding that can take into account the words before and after a word to determine its meaning in context. The BERT model structure is shown in Fig. 2, and the model composition is similar to those of GPT and ELMO. The Chinese BERT model is typically obtained through unsupervised task training on a large number of general-purpose corpora, and it can learn a better feature representation of words and be used directly in downstream tasks. Texts in fields such as biomedicine have a very different structure and word distribution than ordinary texts in general domains, and they contain many long-tailed terms. Therefore, a general domain-based BERT model is unsuitable for medical texts. This paper uses the MC-BERT model from the Chinese medical field to perform word embedding operations on the training data to better learn the medical texts' content information. The structure of the MC-BERT model is the same as that of the BERT model, but different pretraining methods and pretraining corpora are used. Among MC-BERT pretraining approaches, one is mask prediction for medical entities, which only masks medical-related words. This approach replaces 15% of the medical-related words in the Chinese pretraining corpus with [Mask]; 80% of these selected medical words are replaced normally, 10% are replaced with another word, and the last 10% are kept constant for prediction of the masked words. The second pretraining method is "next-sentence prediction," which selects two sentences in the correct order from the same Chinese medical corpus document as positive samples and then randomly selects sentences from different documents to be added after the first sentence as negative samples. The former task focuses on the information between words, and the latter obtains the information between sentences. The integration of these two kinds of information during pretraining can make the word embedding have a better expression effect.

### BiLSTM models

Long short-term memory (LSTM) is a type of recurrent neural network (RNN) model. In comparison to the traditional cyclic RNN structure, LSTM adds three gate structures: an input gate, forget gate, and output gate; it can extract more useful information from neurons. In addition, the LSTM model can effectively solve the gradient disappearance and gradient explosion problems of long text sequences in the training process. The calculation process of the neurons in LSTM is shown in Formulas (1)–(6).

1 $${f}_{t}=\sigma \left({w}_{f}\cdot {h}_{t-1}+{u}_{f}\cdot {x}_{t}+{b}_{f}\right)$$

2 $${i}_{t}=\sigma \left({w}_{i}\cdot {h}_{t-1}+{u}_{i}\cdot {x}_{t}+{b}_{i}\right)$$

3 $${\widetilde{c}}_{t}=\mathit{tan}h\left({w}_{c}\cdot {h}_{t-1}+{u}_{c}\cdot {x}_{t}+{b}_{c}\right)$$

4 $${c}_{t}={f}_{t}\odot {c}_{t-1}+{i}_{t}\odot {\widetilde{c}}_{t}$$

5 $${o}_{t}=\sigma \left({w}_{o}\cdot {h}_{t-1}+{u}_{o}\cdot {x}_{t}+{b}_{o}\right)$$

6 $${h}_{t}={o}_{t}\odot \mathrm{tan}h\left({c}_{t}\right)$$

where ${f}_{t}$, ${i}_{t}$, ${h}_{t}$, ${o}_{t}$, and ${c}_{t}$ represent the forget gate, input gate, hidden layer, output gate, and current cell state, respectively. ${w}_{i},{w}_{f},{w}_{c}$ and ${w}_{o}$ each indicate the weight corresponding to the previous hidden layer ${h}_{t-1}$; ${u}_{i}$, ${u}_{f}$, ${u}_{c}$, and ${u}_{o}$ represent the weights corresponding to the current input vector ${x}_{t}$; and ${b}_{i}$, ${b}_{f}$, ${b}_{c}$, and ${b}_{o}$ indicate the relevant bias vectors. ${\widetilde{c}}_{t}$ is the new candidate state vector. $\sigma$ is the dot product operation, and ⊙ is the sigmoid activation function.

Bidirectional LSTM involves applying a forward and reverse LSTM network to each training text sequence separately, with the two LSTM networks connected to the same output layer. As a result, information in the text can be obtained from both the forward and backward directions, and semantic dependencies of longer distances can be better captured at the sentence level [26].

### CNN models

The convolutional neural network (CNN) model has a convolution layer and a pooling layer, which gives the CNN a good ability to select local features. It can also capture the local semantic relationship between words in a sentence and reduce the dimension of features. Although the CNN was designed to extract image features, it has increasingly been used in natural language processing tasks such as named entity recognition in recent years [27]. In the convolution layer, the text features are subjected to convolution operations through multiple convolution kernels of different sizes, and multiple convolution kernels can be efficiently calculated in parallel, which can further improve the calculation efficiency of feature vectors. The pooling layer extracts the representation of the most important features in the convolutional layer using the max pooling operation, resulting in the text feature vector based on the CNN layer.

### Multihead self-attention

Attention mechanisms are widely used in deep learning-based natural language processing tasks. A study has proposed a self-attention mechanism (Self-Attention) that is combined with BiLSTM and applied to the task of named entity recognition. When extracting text feature information, recurrent neural network models such as RNN and LSTM cannot fully account for the importance of relevant characters in an entire sentence. Even BiLSTM will not obtain much important information in long-distance text. The introduction of a self-attention mechanism can effectively solve the problem of text data time series correlation. To further extract the interactive representation of a text sequence in the text, the multihead self-attention mechanism can obtain semantic feature information from multiple levels and perspectives and obtain the interactive representation of the text sequence.

In all attention mechanisms, there is a task-related query vector $Q$. In addition to the query vector $Q$, the self-attention mechanism adds key-value pairs $K$ and $V$ as matrices. These three matrices are obtained by linear transformation of the weight matrix corresponding to the input sequence, and the dimensions are all ${d}_{k}$, so the three vector matrices $Q$, $K$ and $V$ contain the relevant information of the input features.

In the multihead self-attention mechanism, each self-attention head is also called a parallel computing head. These heads capture the unique feature information of each character in the text sequence in different representation subspaces through multiple independent attentional mechanism calculations; each focuses on a different part of the input. In the multihead self-attention mechanism, the three vector matrices $Q$, $K$ and $V$ require multiple independent linear transformations; that is, they need to be multiplied by multiple different weight matrices W. Therefore, the three vector matrices $Q$, $K$ and $V$ in the multihead self-attention mechanism require multiple mutually independent linear transformations; i.e., they need to be multiplied by multiple different weight matrices W. If this process must be iterated a certain number of times, then the self-attention mechanism that uses the method of scaling the dot product as the scoring function is represented by Formula (7).

7 $$Attention\left(Q{W}^{Q},K{W}^{K},V{W}^{V}\right)=softmax\left(\frac{{Q{W}^{Q}\left(K{W}^{K}\right)}^{T}}{\sqrt{{d}_{k}}}\right)V{W}^{V}$$

where ${Q\in R}^{n\times {d}_{k}}$, ${K\in R}^{m\times {d}_{k}}$ and ${V\in R}^{m\times {d}_{k}}$ are the vectorized sequences obtained after the linear transformation of the input sequence. ${W}^{Q}$∈${R}^{{d}_{k}\times {d}_{k/h}}$, ${W}^{K}\in {R}^{{d}_{k}\times {d}_{k/h}}$ and ${W}^{V}\in {R}^{{d}_{k}\times {d}_{k/h}}$ denote the corresponding parameter weight matrices, and $softmax$ is a column normalization function. The multihead self-attention mechanism combines these h self-attention mechanisms, and its calculation process $\mathrm{MultiHeadAttention}$ is shown in Formulas (8)–(9).

8 $$MultiHeadAttention=Concat\left({head}_{i},\ldots ,{head}_{h}\right){W}^{O}$$

9 $${head}_{i}=Attention\left({QW}_{i}^{Q},{KW}_{i}^{K},{VW}_{i}^{V}\right)$$

where $${head}_{i}$$ repreSru

sents the ith head in the multihead self-attention mechanism, and $\mathrm{Concat}$ represents the concatenation operation. ${W}^{O}$∈${R}^{{d}_{k}\times {d}_{k}}$ is the weight matrix, which changes linearly after combining multiple heads.

### CRF models

Conditional random fields (CRFs) can be used to predict the output in the correct order of the labels by using their constraint relations to ensure the soundness of the entity label output results. Because the models in this paper are all based on the CRF layer output, the scoring function can be defined as in Formula (10).

10 $$score\left(X,y\right)={\sum }_{i=1}^{n}{A}_{{y}_{i},{y}_{i+1}}+{\sum }_{i=1}^{n}{P}_{i,{y}_{i}}$$

where $X$ is the input text sequence $\left({x}_{1},{x}_{2},{x}_{3},\ldots ,{x}_{n}\right)$, ${A}_{i,j}$ and $P$ are the transition matrices and observation matrices, respectively, and the scoring function is the sum of the two matrices. y is the label sequence of the predicted output. As shown in Formula (11), the conditional probability $P\left(y|X\right)$ of $y$ under a given $X$ can be calculated using the scoring function.

11 $$P\left(y|X\right)=\frac{exp\left(score\left(X,y\right)\right)}{{\sum }_{\widetilde{y}\in {Y}_{X}}exp\left(X,\widetilde{y}\right)}$$

where ${Y}_{X}$ represents all possible label sequences for a given sentence and the loss function is defined by Formula (12).

12 $$L=-\sum_{i=0}^{N}logP\left({Y}_{i}|{X}_{i}\right)$$

Following the completion of training, the label sequence ${y}^{*}$ obtained when the scoring function reaches its maximum value can be calculated using Formula (13).

13 $${y}^{*}={argmax}_{\widetilde{y}\in {Y}_{X}}sorce\left(X,\widetilde{y}\right)$$

### Algorithm description

Inputs: One sequence of $k$ text characters $W=[{w}_{1},{w}_{2},\ldots ,{w}_{n}]$ is entered at a time. (W represents the corresponding word in the sentence, and $\mathrm{n}$ denotes the maximum length of the input sentence).

Outputs: The hybrid neural network model produces the output label sequence $Y=[{y}_{1},{y}_{2},\ldots ,{y}_{n}]$ from the input text character sequence $W$ ($y$ is the label that corresponds to the word).

Step 1: Word embedding

Following the word embedding process of MC-BERT, the word vector representation ${\mathrm{V}\in R}^{k\times n\times t}$ is obtained for the input character sequence $\mathrm{W}$ ($t$ is the dimension size of the self-attention head in BERT, usually 768).

Step 2: Downstream models for feature extraction

1. The feature vector matrices ${\mathrm{V}}_{\mathrm{B}}\in {\mathrm{R}}^{\mathrm{h}\times \mathrm{z}}\mathrm{ and }{\mathrm{V}}_{\mathrm{C}}\in {\mathrm{R}}^{\mathrm{r}\times \mathrm{j}\times \mathrm{z}}$ are obtained by feeding the word vector representation $\mathrm{V}$ into the BiLSTM and CNN models, respectively($\mathrm{h},\mathrm{z},\mathrm{r},\mathrm{j}$ represent the corresponding vector dimension values of BiLSTM and CNN);
2. The obtained ${\mathrm{V}}_{\mathrm{B}}\in {\mathrm{R}}^{\mathrm{h}\times \mathrm{z}}\mathrm{ and }{\mathrm{V}}_{\mathrm{C}}\in {\mathrm{R}}^{\mathrm{r}\times \mathrm{j}\times \mathrm{z}}$ are summed according to a certain dimension to obtain the vector matrix ${\mathrm{V}}_{\mathrm{C}}\in {\mathrm{R}}^{\mathrm{m}\times \mathrm{z}}$, and ${\mathrm{V}}_{\mathrm{C}}$ will be put into the multihead self-attention mechanism (MHA) to further obtain the vector matrix ${\mathrm{V}}_{\mathrm{C}}\in {\mathrm{R}}^{\mathrm{v}\times \mathrm{z}}$ ($\mathrm{m},\mathrm{v}$ represent the vector matrix dimension values of the corresponding MHA).

Step 3: Decoding features into output labels

The CRF layer receives the MHA mapped vector matrix ${\mathrm{V}}_{\mathrm{C}}\in {\mathrm{R}}^{\mathrm{v}\times \mathrm{z}}$ and decodes it to produce the NER output label sequence $Y=[{y}_{1},{y}_{2},\ldots ,{y}_{n}]$ (y indicates the label corresponding to the word).

Step 4: Hyperparameters adjustment

The learning rate $\mathrm{\alpha }$, dropout and other hyperparameters of the downstream model training are updated independently, and then the execution steps Step 2 and Step 3 are repeated to train the hybrid neural network model in this paper and return the results. According to the returned results, the relatively optimal $\mathrm{\alpha }$, dropout and other parameter values are selected.

## Experiments

### Experimental parameter setting and evaluation metrics

In this work, we use three metrics, Precision (P), Recall (R) and F1 score, to evaluate the effectiveness of the model named entity recognition. To ensure a fairer experimental comparison, the model uses the same parameter settings with the exception of a few special settings, as shown in Table 1. The AdamW optimizer is used in all experimental models to prevent overfitting. The AdamW optimizer corrects the error of weight decay in the Adam optimizer, which makes the experimental results more accurate [28].

**Table 1:** Model parameter settings

Parameters	Value
LSTM vector dimension	100
Text batch size	32
Learning rate	5e−5
Maximum sentence length	128
Dropout	0.9
Training batch	50

### Dataset representation

This paper uses common datasets of Chinese EMRs, i.e., CCKS-2017 [http://www.sigkg.cn/ccks2017/?page_id=51] and CCKS-2019 [http://www.sigkg.cn/ccks2019/?page_id=62], and the cEHRNER Chinese EMR dataset from the new Chinese Biomedical Language Understanding Evaluation Benchmark (ChineseBLUE) published by Ali's group in the MC-BERT paper. We further process each data item in the original EMR to better fit the training model. For example, the item “右腕疼痛2小时。B-BODYI-BODYB-SYMPTOMI-SYMPTOMOOOO” is adjusted to “右 B-BODY”, “腕 I-BODY”, “疼 B-SYMPTOM”, “痛 I-SYMPTOM”, “2 O”, “小 O”, “时 O”, “。 O”, etc., corresponding to multiple lines of data, where “O” denotes other entities and the words are separated from the labels by a space. “.” or “。” is used as a cut between sentences, and they are separated with a line break. When text is input to the BERT model for word embedding, each data point is preceded by the “CLS” character, and the “SEP” character is added in the middle of a sentence to connect the two sentences.

Since CCKS officially only publishes a training dataset and a test dataset for the training task, we divide these two datasets into a training set, a validation set and a test set in a certain ratio. The entities in the CCKS-2017 EMRs dataset are roughly divided into five categories, and the distribution of entities in the training set, validation set and test set is shown in Table 2, where “BODY”, “TREATMENT”, “SIGNS”, “CHECK”, and “DISEASE” indicate the body part, treatment, symptoms, examination and disease of the patient in the EMR text, respectively. The entities in the CCKS-2019 EMR dataset are also broadly divided into 5 categories, where “LAB” denotes components and “MEDICINE” indicates medical drugs. Its entity distribution is shown in Table 3. The cEHRNER Chinese EMRs dataset includes 914 training sets, 44 validation sets and 41 test sets. The entities in this dataset are roughly divided into 6 categories, where "Operation" is an entity related to the surgical treatment of patients and the distribution of the number of entities in the 6 categories is shown in Table 4.

**Table 2:** Entity distribution in the CCKS-2017 dataset

CCKS-2017	Body	Treatment	Signs	Check	Disease
Train	8458	861	6174	7588	549
Dev	1069	113	811	923	87
Test	1150	72	817	987	85

**Table 3:** Entity Distribution in the CCKS-2019 Dataset

CCKS-2019	Body	Treatment	LAB	Check	Medicine
Train	6777	840	861	3937	1472
Dev	799	143	89	535	199
Test	841	46	229	699	151

**Table 4:** Entity Distribution in the cEHRNER Dataset

cEHRNER	Disease	Operation	Body	Medicine	Symptom	Check
Train	3824	946	5623	1646	2095	2002
Dev	173	52	252	84	78	110
Test	149	43	220	72	88	60

#### Comparison of NER experiments on different Chinese pretraining models

To compare the effectiveness of different Chinese BERT models for named entity recognition on EMR datasets, this paper uses different versions of BERT's pretrained models. These include the Chinese BERT model published by Google, the RoBERT model [29], the RoBERT-WWM model published by the IFLY Laboratory of Harbin Institute of Technology [30], and the MC-BRET model used in this paper based on the biomedical field. The NER tasks were performed on three different EMR datasets, CCKS-2017, CCKS-2019, and cEHRNER. The experimental results are shown in Tables 5, 6 and 7. The bold data in all tables represents the maximum value of the column.

**Table 5:** NER results of different pretraining models on CCKS-2017

BERT models	Precision	Recall	F1
BERT-CRF	89.89	94.02	91.91
RoBERT-CRF	89.57	92.83	91.17
RoBERT_WWM-CRF	91.00	**94.66**	92.80
MC_BERT-CRF	**92.03**	93.99	**93.00**

**Table 6:** NER results of different pretraining models on CCKS-2019

BERT models	Precision	Recall	F1
BERT-CRF	80.99	**86.81**	83.80
RoBERT-CRF	80.43	86.25	83.24
RoBERT-WWM + CRF	82.31	85.58	83.92
MC_BERT-CRF	**84.23**	85.43	**84.43**

**Table 7:** NER results of different pretraining models on cEHRNER

BERT models	Precision	Recall	F1
BERT-CRF	89.02	89.64	89.32
RoBERT-CRF	90.13	90.87	90.5
RoBERT_WWM-CRF	90.21	91.14	90.67
MC_BERT-CRF	**91.08**	**91.67**	**91.37**

From the results of the experimental data in Tables 5, 6 and 7, there is a difference in the effectiveness of NER based on different pretrained BERT models on the three EMR datasets. Using the same CRF as in feature decoding, the MC-ERT + CRF model achieved the best F1 scores of 93.00%, 84.43%, and 91.37% for the three dataset species. The MC-BERT + CRF model also has higher accuracy and recall than most other Chinese pretrained BERT models because the MC-BERT model uses the whole-entity masking strategy to mask medical entities. The whole-span masking strategy is used to mask phrases related to medical entities based on the BERT model, so it can better identify medical-related entities. Therefore, the medical-based MC-BERT model has a significant improvement over other BERT models for the NER task of Chinese EMRs.

#### Experimental comparison of hybrid model NER

We compared the experimental results of other literature models and different BERT-based network models on three datasets, CCKS-2017, CCKS-2019, and cEHRNER, to verify the efficacy of various downstream models for named entity recognition under the medical MC-BERT-based model. After conducting five replicated experiments for each model on the same experimental setup and taking the best results from among them, the final experimental results are shown in Tables 8, 9 and 10.

**Table 8:** Results of different NER models on CCKS-2017

Models	Precision	Recall	F1	Cost time
BiLSTM-CRF	89.47	89.06	89.26	6 h 41 m
RD-CNN-CRF [31]	90.63	92.02	91.32	5 h 56 m
ELMO-BiLSTM-CRF [32]	91.48	93.92	92.66	5 h 41 m
BERT-BiLSTM-CRF	92.03	94.46	93.23	4 h 35 m
BERT-WWM + BiLSTM + CRF [33]	92.24	94.74	93.47	4 h 46 m
MC_BERT-BiLSTM-CRF	92.25	94.98	93.60	4 h 22 m
MC_BERT-BiLSTM-MHA-CRF	92.62	94.89	93.74	4 h 33 m
MC_BERT-BiLSTM-CNN-CRF	92.73	95.11	93.90	4 h 41 m
MC_BERT-BiLSTM-CNN-MHA-CRF	**93.04**	**95.43**	**94.22**	4 h 57 m

**Table 9:** Results of different NER models on CCKS-2019

Models	Precision	Recall	F1	Cost time
BiLSTM + CRF	81.11	80.47	80.79	6 h 23 m
RD + CNN + CRF [31]	81.87	82.03	81.95	5 h 43 m
ELMO-BiLSTM-CRF [32]	82.31	81.89	81.10	5 h 33 m
BERT-BiLSTM-CRF	82.09	87.32	84.62	4 h 26 m
BERT-wwm + BiLSTM + CRF [33]	82.47	87.42	84.87	4 h47 m
MC_BERT-BiLSTM-CRF	83.10	87.42	85.20	4 h 13 m
MC_BERT-BiLSTM-MHA-CRF	83.24	**87.93**	85.52	4 h 39 m
MC_BERT-BiLSTM-CNN-CRF	83.04	87.77	85.34	4 h 32 m
MC_BERT-BiLSTM-CNN-MHA-CRF	**84.90**	87.67	**86.27**	4 h 48 m

**Table 10:** Results of different NER models on cEHRNER

Models	Precision	Recall	F1	Cost time
BiLSTM-CRF	84.72	85.14	84.92	5 h 18 m
RD + CNN + CRF [31]	86.36	87.23	86.79	5 h
ELMO-BiLSTM-CRF [32]	87.54	87.33	87.43	4 h 37 m
BERT-BiLSTM-CRF	89.35	89.96	89.65	3 h 31 m
BERT-wwm + BiLSTM + CRF [33]	90.17	90.46	90.31	3 h 54 m
MC_BERT-BiLSTM-CRF	91.69	92.14	91.91	3 h 25 m
MC_BERT-BiLSTM-MHA-CRF	92.11	92.32	92.21	3 h 36 m
MC_BERT-BiLSTM-CNN-CRF	92.24	92.54	92.38	3 h 47 m
MC_BERT-BiLSTM-CNN-MHA-CRF	**92.78**	**92.88**	**92.82**	3 h 56 m

In obtaining the experimental results of the three datasets in Tables 8, 9 and 10, the RD + CNN + CRF model uses a residual expansion convolutional neural network with dictionary features and conditional random fields [31]. Compared with the basic BiLSTM + CRF model, the three evaluation indicators improved, and the three F1 scores increased by 2.06%, 1.16% and 1.87%, respectively. The BiLSTM + CRF model with the integration of pretrained ELMo has a significant improvement in evaluation compared to the BiLSTM + CRF model [32], which indicates that the pretrained ELMo model has a significant improvement in the effectiveness of the deep learning model for named entity categories. The BiLSTM + CRF model based on pretrained BERT and pretrained ELMo further improved accuracy, recall and F1 on the three datasets, and the F1 values of the evaluation results achieved 93.23%, 84.62% and 89.65%, respectively, which are higher than the F1 values of the basic BiLSTM + CRF model by 2.92%, 3.83% and 4.73%, respectively. Compared with BERT, BERT-wwm uses a full-word mask to obtain a comprehensive word vector representation [33]. Therefore, the BERT-wwm + BiLSTM + CRF model has a slight improvement in each evaluation value compared with the BERT + BiLSTM + CRF model.

The BiLSTM + CRF model based on medical MC-BERT improved in all three evaluation metrics compared to the generic pretrained BERT model BiLSTM + CRF, and the F1 values improved to 93.60%, 85.20% and 91.91%. The medical MC-BERT model has greater improvement on the EMR dataset compared to the generic BERT model, which further illustrates the effectiveness of the medical MC-BERT model for mining medical text entities. Compared with the BiLSTM + CRF model based on MC-BERT, the effect of the hybrid model with the integration of a convolutional neural network (CNN) or multihead self-attention mechanism (MHA) in the downstream model also has a corresponding improvement in the evaluation metrics. The MC-BERT-based BiLSTM + MHA + CRF and BiLSTM + CNN + CRF network structures can reach F1 scores of 93.74%, 93.90%, and 92.21% and 85.52%, 85.34%, and 92.38% on these three datasets, respectively, which are better than the scores of the MC-BERT + BiLSTM + CRF model. This indicates that the integration of CNN and MHA can effectively capture the local features of text and the significance of different characters in a sentence, which in turn can improve the model to some extent. From the results in the three tables, it is clear that any downstream model based on an MC-BERT pairing will yield better results than those in Tables 4, 5 and 6, which use CRF decoding alone. The hybrid neural network model MC-BERT + BiLSTM + CNN + MHA + CRF with both CNN and MHA integrated into the medical MC-BERT-based BiLSTM + CRF improved the results in terms of accuracy, recall, and F1 value compared to the results of integrated the CNN model and MHA model structure alone in BiLSTM + CRF. This model can obtain the best F1 values of 94.22%, 86.27% and 92.82% on the three EMR datasets, which are 0.62%, 1.07% and 0.91% higher, respectively, than the F1 values of the MC-BERT + BiLSTM + CRF model without the integration of the CNN and MHA models. The results in these three tables show that the hybrid neural network model based on MC-BERT proposed in this paper achieves good evaluation results.

Our proposed MC-BERT + BiLSTM + CNN + MHA + CRF model has a significantly shorter training time than the basic BiLSTM + CRF model. In addition, the training time of our MC-BERT + BiLSTM + CRF is slightly lower than that of the BERT + BiLSTM + CRF model because MC-BERT is obtained by further pretraining on the medical text corpus based on the BERT model; therefore, the convergence speed for medical text recognition is slightly faster. However, after integrated the CNN and MHA models to MC-BERT + BiLSTM + CRF, the network model structure becomes complex, so there is a corresponding increase in training time.

### Analysis of the effect of different models on the recognition of the same electronic medical record instance

**Fig. 3:**
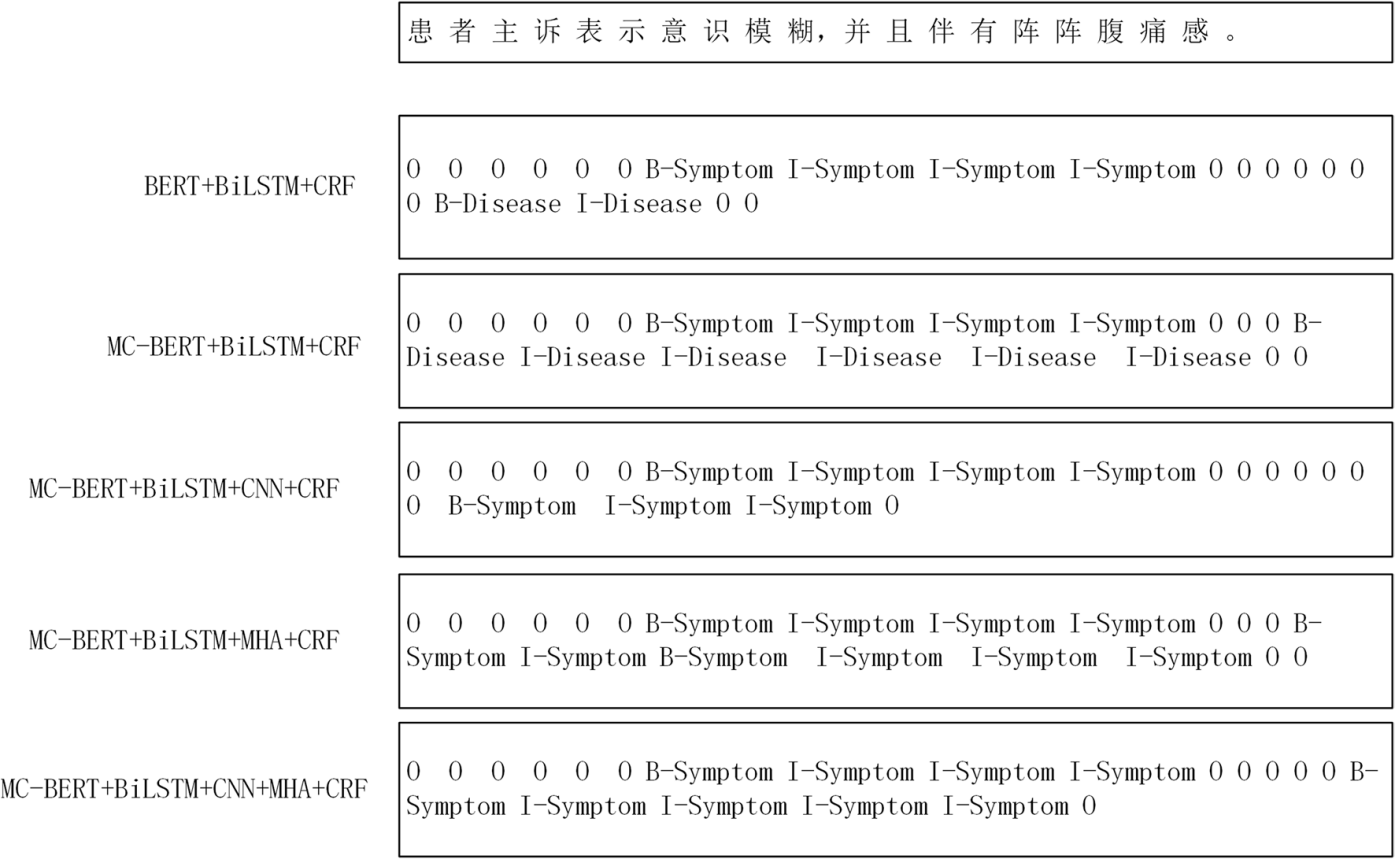
The effect of different models on the recognition of the same electronic medical record instance

For the same Chinese EMR text, the entity effects recognized by different pretraining models are different, and the specific effects are shown in Fig. 3.

As can be seen in Fig. 3, in one Chinese EMR text, “患者主诉表示意识模糊，并且伴有阵阵腹痛感。[The patient complained that he felt a blurring of consciousness accompanied by bursts of abdominal pain sensation.]”, “阵阵腹痛 [bursts of abdominal pain]” is the clinical manifestation of the patient's symptoms in the medical text. However, the BERT + BiLSTM + CRF model incorrectly identifies it as a disease-like entity and only recognizes “腹痛[abdominal pain]” in it. After replacing the BERT model with the medical MC-BERT, the overall “伴有阵阵腹痛 [accompanied by bursts of abdominal pain]” can be identified as a class entity of “Disease”. The reason is that MC-BERT co-adds boundary words around medical entities as entities for training in the pretraining phase, so the MC-BERT-based NER model can identify a more comprehensive set of entities. With the integration of CNN models into the downstream BiLSTM + CRF model, we were able to accurately identify “腹痛感 [abdominal pain sensation]” as a symptom-related entity, although not all entities were identified completely based on the local contextual feature information extracted. With the integration of the MHA model into the downstream model, all of “伴有阵阵腹痛感 [accompanied by bursts of abdominal pain sensation]” was identified as a symptom entity from the multilevel semantic features, but the unrelated “伴有 [accompanied by]” was also jointly identified as a symptom entity.

We propose that the MC-BERT + BiLSTM + MHA + CNN + CRF model not only recognizes “腹痛感 [abdominal pain sensation]” as a symptom-related entity but also successfully recognizes “阵阵[bursts]”,, which is used to denote the degree adverb associated with “腹痛感 [abdominal pain sensation]”. As the results show, the entities identified by combining the hybrid neural network model are more accurate. The successful recognition is due to the all-entity masking strategy of MC-BERT during pretraining and the adequate extraction of text feature information by the downstream hybrid neural network. Therefore, compared with those of the BERT + BiLSTM + CRF-based model, the medical-related entities identified by the MC-BERT + BiLSTM + MHA + CNN + CRF model that we propose in this paper are more comprehensive and rigorous.

### Effect of different ratios of entities on the experimental results and analysis

**Fig. 4:**
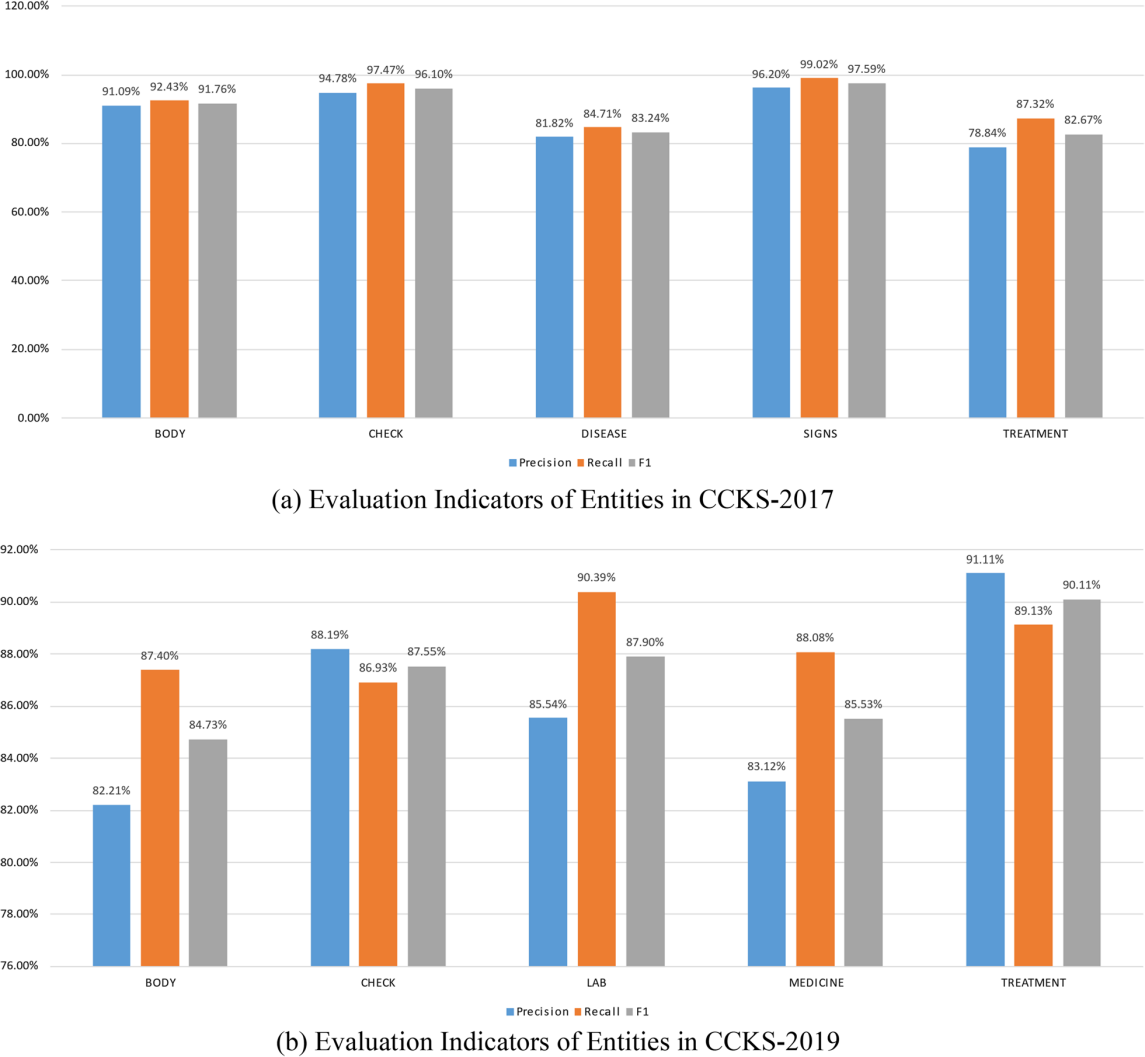
Evaluation values of two different entities in two datasets

The experimental results of all models in this paper are based on the evaluation results at the entity level; that is, the models' final accuracy, recall, and F1 score evaluation values are derived from the evaluation index results of each entity. Therefore, the evaluation results of the individual entities in the dataset have a significant influence on the final results of the whole model. In Fig. 4a, b shows the three evaluation metrics of the entities obtained from testing the BiLSTM + CNN + MHA + CRF hybrid neural network model based on the medical MC-BERT proposed in this paper on two datasets: CCKS-2017 and CCKS-2019.

As shown in Fig. 4, the performance of entity recognition varies between datasets. In Fig. 4a, for the CCKS-2017 dataset, the three evaluations for each entity were similar, with the “BODY”, “CHECK”, and “SIGNS” entities all scoring 91% or higher and the SIGNS entity scoring 96% or higher for all three tests. The other two entities, “DISEASE” and “TREATMENT”, have evaluation values that are less than 90%, and their evaluation performance is not as good as that of the first three. This is caused by the uneven distribution of the numbers of each type of entity across the training and test datasets. Table 2 shows that the numbers of “BODY”, “CHECK”, and “SIGNS” entities are roughly ten times those of the two relatively small entity types DISEASE and TREATMENT and that the final output of the model is jointly determined by the evaluation value of each entity. As a result, if the model fails to accurately predict the majority of entities, it is difficult to improve the individual assessment values of those entities, which has an impact on the model's overall assessment value. However, for a larger number of entities, while there will be a few entities that are not accurately identified, the overall effect of entity assessment will also be better due to the advantage of the larger number. Because a larger number of entities are trained more fully during the training process, the model has a better ability to recognize them and ultimately performs better on the test data.

Since the number distribution of each entity in CCKS-2019 is very uneven in Table 3 and the proportions of entities in the training set and test set are also different, the three assessed values of each entity in Fig. 4b vary, and the corresponding assessed values of different entities are also different. For example, compared to the accuracy rates of the entities “BODY” and “MEDICINE”, which are only 82.21% and 83.12%, respectively, the accuracy of the entity “TREATMENT” is 8.9% and 7.99% higher, respectively, and the other evaluated values of “BODY” and “MEDICINE” are lower than the evaluated values corresponding to the other three entities. Additionally, the precision and recall of the three entities “BODY”, “LAB” and “MEDICINE” differ significantly, which results in their F1 scores being lower than those of the other two entities. This results in a more mediocre performance of each overall assessment value for the CCKS-2019 dataset.

From the statistical results of the number of entities in Tables 2 and 3 and the effect of named entity identification for each entity on the two datasets in Fig. 4, the uneven proportion of the number of entities in the EMR text leads to differences in the entity assessment metrics, and the entities with lower assessment values will have an impact on the final NER assessment results. The above considerations also apply to the entities in the cEHRNER dataset, which are not further analyzed here. Therefore, a balanced proportion of the number of entities in Chinese EMRs is essential for their named entity identification.

## Conclusions and future scope

In this study, we introduced a hybrid neural network method based on medical MC-BERT to extract entities from Chinese EMRs. The method first uses a Chinese medical-related MC-BERT model as the word vector representation of the EMR text; it incorporates a bidirectional LSTM and CNN model to capture the long-term dependencies and local feature information of a sentence, then uses the MAH model to obtain multilevel semantic features in the sentence, and finally uses CRF decoding to obtain entities. The experimental results show that our method is applicable to extracting entities related to Chinese medical texts in Chinese EMRs. Finally, this paper analyzes the effect of different proportions of entities on the experimental results when using a hybrid neural network model for the named entity recognition task.

In the future study, we will attempt to further improve the performance of the named entity recognition model by incorporating additional Chinese medical-related word vector features into the pre-trained model, such as Chinese medical word radicals and pinyin character vectors, etc.

## Data Availability

Some or all data that support the findings of this study are available from the corresponding author upon reasonable request.
